# Construction Notes

**Published:** 1896-01-04

**Authors:** 


### CONSTRUCTION NOTES.

### New Cottage Hospital at Tilbury.

The foundation-stone of this building, the outcome of Mr. Passmore Edwards' generous gift of £2,000, was laid a short time since. The following is a description of the building: The entrance is placed in the centre of the front and communicates with the hall, from which a well-lighted corridor runs right and left to the wards. The accommodation consists of a surgeon's room, 15 by 12 feet, with dispensary adjoining; matron's rooms, with nurses' sitting-room adjoining, two single wards, and two three-bed wards, each 21 feet by 15 feet. A building two storeys high is placed in the rear of the central hall, containing kitchen and offices on the ground floor, and matron's, nurses', and servants' bed-rooms on the first floor. This block is connected with the main building by means of a cross-ventilated lobby, every effort being made to separate the residential part of the building from the hospital proper. Each wing is provided with a sanitary pavilion, connected with the main corridor by a cross-ventilated lobby, and the building is so arranged that one half can be used for males, and the other half for females. Each pavilion contains a bath-room, a lavatory, and sink, &c. The building, which is to be of an attractive form, is faced with red brick and Bath stone dressings. The architect is Mr. Rowland Plumbe, and Mr. J. Brown, of Grays, is the builder; the contract price for the hospital, exclusive of foundations, is for £1,892.

### Northallerton Cottage Hospital, Dundas Memorial Wing.

The new wing in connection with this cottage hospital has now been opened; it has been dedicated to the memory of the late Hon. J. C. Dundas, and to the use of the North Riding Rural District Nursing Association. Besides the extension, there has been a great alteration and complete remodelling of the long narrow wing which juts out at right angles from the back of the hospital which faces the street. The entirely new part consists of a three-storeyed brick building, added to and crossing the end of the wing. It is constructed on the following plan: On the base there is a spacious laundry with many special appliances; behind this is a back kitchen, and passing out into the north side of the new buildings on the base is a room to store luggage, an airy pantry, and larder. Ascending the new stairs on the same side (north) is a fair-sized landing, on the north side of which is a spacious bed-room, with three beds for nurses, and a similar bed-room on the south side. On the story above are two similar bed-rooms, one for night nurses and another for servants. With regard to the alterations of the old narrow wing that connects the hospital proper to the new nurses' quarters, one is that the old larder has been converted into a bath-room and lavatory, whilst the former pantry has been converted into a surgery, divided from the passage by a glass partition. Considerable alterations also have been effected in the nurses' accommodation, and very comfortable bed and sitting rooms have now been provided for their greater comfort. Mr. Hodgson Fowler, of Durham, was the architect of this extension work.

### Murthly Asylum, Perth; Extensive Additions.

An extensive addition to Murthly Asylum has been for some time in course of construction, and is now almost completed. Two new convalescent houses—one for male and the other for female patients—are being built by the district Board of Lunacy to relieve the overcrowding previously experienced at the asylum. The house for female patients, which is constructed to accommodate forty-eight, and four nurses, has been built from plans prepared by Mr. David Smart, of Perth. It is a square three-storey building in the front, with a two-storey and a one-storey addition at the back. The frontage consists of two gables and a recessed centre, which is occupied by a broad glass verandah. It faces the south, and all the sitting-rooms get the benefit of the sun. The architect has adopted a domestic style of architecture, with gables, dormer windows, and bow windows. On the ground floor are four public rooms, and a commodious entrance hall for the forty-eight patients, which acts as a sitting-room. A bagatelle board and a piano have been provided, and everything supplied which can tend to suggest a home-like effect upon the inmates. The other apartments on the ground floor are the dining-room and the kitchen, which is conveniently placed immediately behind the dining-room. The two upper storeys consist principally of four large bedrooms for patients, and bedrooms and sitting-rooms for the nurses, and a general bath-room. Each of the four bed-rooms for the patients contains twelve beds. Off the patients' bed-rooms is the nurse's private bed-room, communicating with a glass door with open panels; speaking tubes are placed in the nurses' rooms, therefore the staff are in immediate communication with each other. The lighting is effected by means of electricity, the wards are heated throughout with hot-water pipes, and fire hydrants are placed on all landings.

### Birmingham Eye Hospital.

The development now completed at this institution is indeed a notable and an absolutely necessary one. Under the old conditions the children and women were treated on the same side of tha hospital, and this arrangement has great disadvantages. Moreover, there were strong medical reasons for the erection of a new wing. The governors adopted the designs for this by Mr. Henry F. Talbot. The broad scheme of work is the provision of a separate department for children and better accommodation for nurses and servants, also a re-arrangement of the lavatories, &c., and of some of the old wards. Further, the out-patients' department has been enlarged by the provision of additional minor operating and dark rooms and a handsome large hall for men. The children's department is all placed on the one floor level. It consists of day-room and serving kitchens, two large and two small wards, linen and bath rooms, &c. The upper floor of the new buildings, which in architectural construction is of the nature of a continuation of the original block, is entirely set apart for the accommodation of nurses and servants. The lavatories &c., on each floor are contained in a separate building at the rear, approached by disconnecting lobbies. Messrs. Harley and Sons, of Smethwick, are the contractors. The total cost of the work is about £5,000, nearly the whole of which amount has already been subscribed.

